# Comprehensive and integrated district health systems strengthening: the Rwanda Population Health Implementation and Training (PHIT) Partnership

**DOI:** 10.1186/1472-6963-13-S2-S5

**Published:** 2013-05-31

**Authors:** Peter C Drobac, Paulin Basinga, Jeanine Condo, Paul E Farmer, Karen E Finnegan, Jessie K Hamon, Cheryl Amoroso, Lisa R Hirschhorn, Jean Baptise Kakoma, Chunling Lu, Yusuf Murangwa, Megan Murray, Fidele Ngabo, Michael Rich, Dana Thomson, Agnes Binagwaho

**Affiliations:** 1 Partners In Health/Inshuti Mu Buzima, Rwinkwavu, Rwanda; 2 Division of Global Health Equity, Brigham and Women’s Hospital, Boston, 02115, USA; 3 Harvard Medical School, Boston, 02115, USA; 4 The Bill & Melinda Gates Foundation, Seattle, 98109, USA; 5 School of Public Health, National University of Rwanda, Kigali, Rwanda; 6 National Institute of Statistics of Rwanda, Kigali, Rwanda; 7 Ministry of Health, Republic of Rwanda, Kigali, Rwanda; 8 Geisel School of Medicine, Dartmouth College, Hanover 03755, USA; 9 Partners In Health, Boston, 02115, USA

## Abstract

**Background:**

Nationally, health in Rwanda has been improving since 2000, with considerable improvement since 2005. Despite improvements, rural areas continue to lag behind urban sectors with regard to key health outcomes. Partners In Health (PIH) has been supporting the Rwanda Ministry of Health (MOH) in two rural districts in Rwanda since 2005. Since 2009, the MOH and PIH have spearheaded a health systems strengthening (HSS) intervention in these districts as part of the Rwanda Population Health Implementation and Training (PHIT) Partnership. The partnership is guided by the belief that HSS interventions should be comprehensive, integrated, responsive to local conditions, and address health care access, cost, and quality. The PHIT Partnership represents a collaboration between the MOH and PIH, with support from the National University of Rwanda School of Public Health, the National Institute of Statistics, Harvard Medical School, and Brigham and Women’s Hospital.

**Description of intervention:**

The PHIT Partnership’s health systems support aligns with the World Health Organization’s six health systems building blocks. HSS activities focus across all levels of the health system — community, health center, hospital, and district leadership — to improve health care access, quality, delivery, and health outcomes. Interventions are concentrated on three main areas: targeted support for health facilities, quality improvement initiatives, and a strengthened network of community health workers.

**Evaluation design:**

The impact of activities will be assessed using population-level outcomes data collected through oversampling of the demographic and health survey (DHS) in the intervention districts. The overall impact evaluation is complemented by an analysis of trends in facility health care utilization. A comprehensive costing project captures the total expenditures and financial inputs of the health care system to determine the cost of systems improvement. Targeted evaluations and operational research pieces focus on specific programmatic components, supported by partnership-supported work to build in-country research capacity.

**Discussion:**

Building on early successes, the work of the Rwanda PHIT Partnership approach to HSS has already seen noticeable increases in facility capacity and quality of care. The rigorous planned evaluation of the Partnership’s HSS activities will contribute to global knowledge about intervention methodology, cost, and population health impact.

## Background

A decade after the 1994 genocide, which claimed the lives of at least 800,000 people and left 2 million homeless, Rwanda’s health system remained fragile across much of the country. Mortality rates, which had skyrocketed in the mid-1990s, did not return to pre-1994 levels until 2005 [1]. In 2005, 57% of the population was living in poverty (defined as 118,000 Rwandan francs or approximately $200, per year), and only 30% of those experiencing an illness or accident reported seeing a medical practitioner [2].

In 2005, the Government of Rwanda (GOR) and the Clinton Foundation invited Partners In Health (PIH) to help rebuild the public health infrastructure in two of the country’s most impoverished rural districts: southern Kayonza and Kirehe. Employing a model of multilevel public sector health system strengthening used by PIH in rural Haiti, the Rwandan Ministry of Health (MOH) and PIH rapidly invested in health facility infrastructure, building or renovating two district hospitals and six health centers; recruited and trained health professionals from the limited available pool; and introduced community-based HIV care integrated with basic primary health care services [3].To bring the lessons of this successful collaboration to scale, the MOH, PIH, and other partners developed a detailed framework for district-level health systems strengthening (HSS) aligned with the World Health Organization (WHO) six building blocks: service delivery, health workforce, information, medicines, financing and governance [4]. This framework was incorporated into the 2009 National Health Sector Strategic Plan (HSSP-II), comprised of seven strategic program areas: institutional capacity, human resources for health, financial accessibility, geographic accessibility, medical products and consumables, service delivery quality, and specialized services and research capacity [5].

### Utilization and health outcomes in Rwanda, 2005-2009

By 2009, the Rwandan government had improved access to primary health services mainly through infrastructure development and expansion of a community-based health insurance program. Geographic access to care was relatively well distributed, with 75% of the nation’s population living within 5 kilometers of a health facility [6]. Although one of the world’s poorest countries, Rwanda created the *Mutuelle de Santé* health insurance program, which provided access to basic primary health care services for 85% of its citizens by 2009 [7]. Nevertheless, there was a marked shortage of trained health care workers with 0.04 generalist physicians and 0.62 nursing professionals per 1,000 people [6]. Due, in part, to increased health care access, health outcomes in Rwanda had improved since 2000 [8]. Under-5 mortality, a useful gauge of health system impact on overall child health [9], fell from 152 deaths per 1,000 live births in 2005 to 103 in 2008 (five-year mortality estimates) [10]. Consistent with patterns in other low- and middle-income countries, child and infant mortality rates declined more steeply than neonatal mortality; from 2001-2005, neonatal mortality was 37 per 1,000 live births annually [1], compared with 28 per 1,000 live births from 2004-2008 [10]. Concurrent with increased uptake of family planning services, the total fertility rate declined from 6.1 in 2005 to 5.5 in 2008. Ninety-six percent of pregnant women received some antenatal care and 45% delivered at a health facility in 2008, an increase from 26% who delivered at a health facility in 2000. Utilization by children was lower, with 35% of children with a fever and 33% of children with diarrhea treated at a health facility [10].

### The Rwandan health system

**Figure 1:**
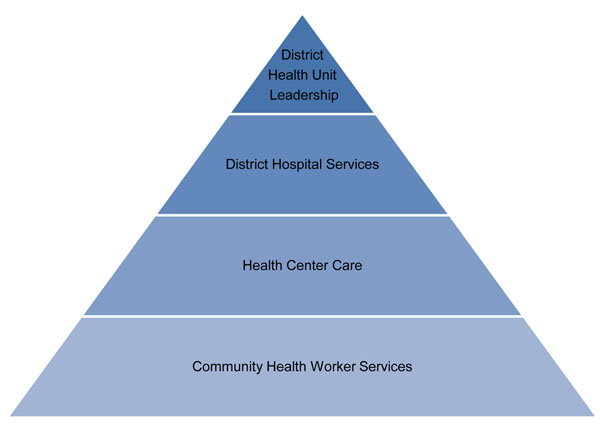
District health structure in Rwanda PHIT intervention area.

Rwanda is divided into 30 districts, and 85% of the country’s 10.8 million people live in rural areas [11]. The architecture of the district health system is typical for a developing country setting (Figure 1). Each district is served by a network of community health workers (CHWs) — three per village — offering health education, basic preventive and curative services, and family planning. CHWs are supported by local health centers, which serve approximately 20,000 people and are staffed by nurses, most of whom have a secondary school education level. Health centers provide vaccinations, reproductive and child health services, acute care, and diagnosis and treatment of HIV, tuberculosis, and malaria. District hospitals, staffed in part by 10-15 generalist physicians, provide more advanced care, including basic surgical services, such as caesarean sections. District pharmacies procure essential medicines and consumables from a central agency and distribute them to all health facilities within the district. District health units are responsible for administrative management of the district health system, while district hospitals are responsible for clinical supervision and monitoring and evaluation of all district health facilities.

### The Rwanda Population Health Implementation and Training (PHIT) Partnership

In 2009, the Rwanda Population Health Implementation and Training (PHIT) Partnership was established with the support of the Doris Duke Charitable Foundation’s African Health Initiative. The PHIT Partnership designed a multidimensional and integrated district-level HSS intervention that aligned with Rwanda’s 2009 National Health Sector Strategic Plan and the WHO Health Systems Framework. The partnership endeavors to improve the capacity and performance of the health system in southern Kayonza and Kirehe districts with targeted financial and technical investments focused on the health centers, coupled with quality improvement (QI) initiatives designed to improve both service delivery and strengthen monitoring and evaluation (M&E) systems. We hypothesize that the intervention will result in demonstrable improvements in population health, exceeding the already considerable health gains being made nationally.

The PHIT Partnership aspires to create a replicable, evidence-based road map for district-level primary health care delivery in rural Africa and to disseminate lessons learned within and beyond Rwanda’s borders. Accordingly, the partnership has developed an integrated model of implementation, operational research, and impact evaluation to facilitate refinement and rigorous evaluation of the intervention.

The PHIT Partnership is a public-private-academic collaboration comprised of six institutions. The Rwandan MOH and PIH lead the implementation and jointly fund the health system in the intervention area. The Ministry of Health sets national health policies and oversees the health management information system (HMIS) and planned national data warehouse, which provide data for both routine M&E and the impact evaluation. Harvard Medical School (HMS) and staff from Brigham and Women’s Hospital (BWH) faculty support evaluation activities, as well as capacity building for M&E and research components of the partnership. The National University of Rwanda School of Public Health (NUR-SPH) takes part in the evaluation team and leads the research capacity building program. Finally, the National Institute of Statistics of Rwanda (NISR) coordinates and implements the demographic and health surveys (DHS) and other key data collection activities, including the national census and the economic living conditions survey used for the impact evaluation.

### Guiding principles of the Rwanda PHIT Partnership

The Rwanda PHIT Partnership is guided by five key principles that we believe are essential elements of successful HSS interventions. First, health systems interventions should be *comprehensive*, as enshrined in the 1978 conference on International Primary Health Care in Almaty, Kazakhstan, through the Alma Ata Declaration [12]. Second, *integrated* interventions that apply a “systems thinking” approach deliver more value than isolated vertical interventions [13]. Third, interventions should be *locally responsive*, reflecting the engagement of local government leaders, civil society, and the community. Fourth, sustainability of a public health approach can only be achieved with efforts to *strengthen institutions*. Fifth, health systems should place a premium on equity by addressing issues of *access*, *quality*, *and cost*.

### Geographic scope of the intervention

**Figure 2:**
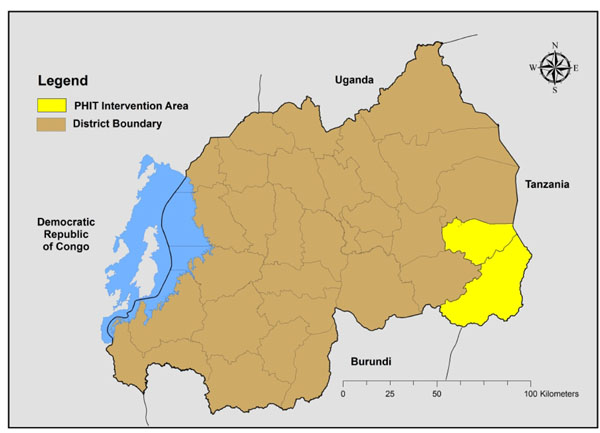
District and PHIT intervention area, Rwanda. Source: Government of Rwanda Ministry of Public Works, Transport and Communications (formerly Ministry of Public Works and Energy), Center for Geographic Information System and Remote Sensing of the National University of Rwanda, National Institute of Statistics or Rwanda. 2005 [http://giscenter.nur.ac.rw/spip.php?rubrique32]. Created by Fabien Munyaneza, 2012.

The Rwanda PHIT intervention area comprises two PIH-supported rural districts — southern Kayonza and Kirehe, which are contiguous over an area of roughly 3000 km^2^ in the Eastern Province of Rwanda (Figure 2). The intervention catchment area comprises 480,000 people, with nearly 2,800 CHWs, 23 health centers, and two district hospitals.

Health and social indicators in the Eastern Province, which includes the PHIT Partnership catchment area, were markedly worse than the rest of the country at the beginning of the project period. In the 2008 interim DHS, southern Kayonza and Kirehe had a total fertility rate of 6.2, compared to 5.6 in other rural areas. The intervention area also had a lower percentage of women completing four standard antenatal care visits (15.6% versus 24.0%). Under-5 mortality remained higher in the intervention area than other rural areas (125.5 vs. 107.9) [10].

Although the two intervention districts are poor, with only 4.4% of households having electricity and the majority of households relying on wood as the main cooking fuel, 78.8% of women and 90.7% of men were employed, predominantly in the agricultural sector [14]. Within southern Kayonza and Kirehe districts, 59.8% of women aged 15-49 had completed some primary education, compared to 62.6% of men [15]. However, due to a strong government commitment to education access, the net primary school attendance ration had reached 87% of children nationally by 2010 [15].

## The Rwanda PHIT intervention

**Figure 3:**
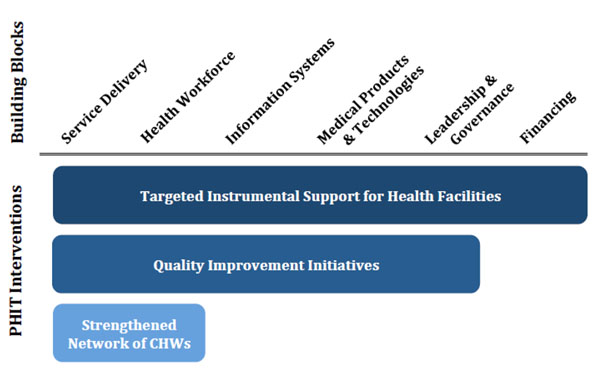
Rwanda PHIT intervention mapped to WHO health system building blocks.

The Rwanda PHIT intervention employs a district-based HSS approach aimed at improving the coordination and quality of care at the health facility and community levels. The intervention aligns Rwanda’s National Health Sector Strategic Plan priority areas with the WHO Health Systems Framework [4] and enhances the existing Rwanda health sector strategy through three main components: capacitation of health facilities through targeted instrumental support, innovative quality-improvement initiatives, and activities aimed at strengthening the existing network of community health workers. Through a focus on facility- and community-level care, with an emphasis on quality, the intervention works to improve service delivery across district-access points. Health centers receive a package of instrumental support that includes targeted financial support, systems improvements, enhanced training and supervision for clinical staff, and continuous quality improvement and data utilization support. The support package is tailored to address facility-specific gaps based on a systematic and participatory analysis. The network of community health workers receives complementary support around supervision, training, and enhanced data collection and use. Core activities are summarized in Table 1. Figure 3 outlines the hypothesized impact of the three intervention components on each of the six WHO building blocks.

**Table 1:** Intervention activities across the different levels of care in the PHIT catchment area

Level	Intervention
Community *	Training of CHWs in primary healthcare modules
	
	Enhanced supervision of CHW performance and data quality
	
	Household register logging CHW activities at household
	
	Support of chronic care patients (HIV, non-communicable disease)
	
	Additional performance-based incentives

Health Center	Facility infrastructure improvements
	
	Financial support for additional staff to reach national Human Resources for Health norms
	
	Community-based health insurance funds to pay inscription and service fees for impoverished patients
	
	Primary care electronic medical record (EMR)
	
	MESH mentorship program to improve quality of care
	
	M&E support for data quality and use
	
	Training of clinical staff

District Hospital **	Facility improvements
	
	Financial support for additional staff to reach national Human Resources for Health norms
	
	Support for district ambulance network
	
	Pharmaceutical procurement for non-essential medications
	
	M&E support for data quality and use

District Health Unit	Mapping of administrative boundaries, health facilities and water sources for planning
	
	Management and planning collaboration
	
	Stock and stock management assistance for district pharmacy

*In southern Kayonza District only

**Supported by PIH independent of PHIT initiative, with exception of M&E support

### Component 1. Targeted instrumental support for health facilities

Without adequate infrastructure — equipment, staffing, financial resources, procurement and information systems, ambulance network, and management capacity — process interventions designed to improve service delivery at health facilities are likely to fail. Therefore, we designed this intervention component to promote a flexible, evidence-based approach to improve health system readiness.

The approach utilizes a health facility survey, which measures capacity in 11 key domains essential to the delivery of quality and effective services: infrastructure, medical equipment, human resources, clinical services, social services, monitoring and evaluation systems, laboratory, pharmacy, infection control, management, and referral systems (Table 2). Following the initial assessment, the PHIT team met with the district leadership and facility staff to identify gaps and allocate financial and technical resources accordingly. Health facility support and its effects are monitored through quarterly re-assessments, the results of which are reviewed jointly with district and facility leadership at quarterly data sharing meetings. Community participation is further emphasized through Community Health Advisory Boards, which ensure that community voices are represented in intervention activities and provide a forum for the project to provide feedback on activities and impact.

**Table 2:** Domains measured by PIH/PHIT facility survey and mapped to WHO health systems building blocks

Domain	Example domain measures	WHO building block
Clinical services	Range of services and availability	Service delivery

Infrastructure	Quality of facility infrastructure for services offered	Service delivery

Social services	Insurance coverage, capacity to provide targeted socioeconomic support	Service delivery, Financial

Referral	Availability of emergency transfer services, communication	Service delivery, Information systems

Monitoring and Evaluation/Data use	Data systems and utilization	Information systems,* Leadership & governance

Medical equipment	Availability of essential equipment for service delivery by specialty, condition of equipment	Medical products and technology

Laboratory	Availability of essential tests, equipment, and supplies	Medical products and technology

Pharmacy	Stock monitoring, essential medicine stock outs, storage and distribution capacity	Medical products and technology

Infection control and waste management	Waste disposal, management of human and medical waste	Service delivery

Human resources	Number of trained staff, staff retention rates	Human resources

Management	Staff supervision, management practices	Leadership & governance

*An additional intervention, targeting the information systems building block, is a web-based primary care electronic medical record (EMR), which is being assessed for feasibility through measured rollout in southern Kayonza.

### Component 2. Quality improvement initiatives

The PHIT intervention emphasizes the improvement of service quality through two main pathways: the Mentorship and Enhanced Supervision of Health Centers (MESH) program and support for M&E systems, focusing on ensuring effective routine monitoring and improved data quality and utilization.

#### Mentorship and Enhanced Supervision of Health Centers (MESH) program

Rwandan health centers are staffed almost exclusively by nurses, who typically receive didactic training and intermittent supervision from district hospital-based physicians and district health officials. The MESH program aims to improve service quality by enhancing the supervision and training of health center nurses while also addressing system-level gaps in quality [16]. MESH is structured around four clinical content areas reflecting national priorities: integrated management of childhood illness (IMCI); HIV; maternal health — including antenatal care, labor and delivery, and postpartum follow-up services; and integrated management of adult illness (IMAI).

MESH assures an adequate knowledge base through didactic training in key content areas and complements this training with ongoing mentorship and supportive supervision. This is achieved through reinforcement of the existing MOH system of health center supervision: increasing the number of available staff dedicated to supervision, improving the technical skills of staff, providing staff tools for clinical and programmatic mentorship, and ensuring more supervision time per health center. Mentors with the MESH program visit each health center for two days every six weeks, conducting structured observation of nurse-delivered care, providing case-based teaching, and working with the health center leadership to identify and address system and quality gaps using continuous QI techniques. Quality of care is assessed through direct observation of patient consultations based on a checklist developed according to national protocols and making use of validated instruments where possible, as in the case of IMCI [^17^].

#### Monitoring and evaluation (M&E) systems support

An integrated system of ongoing routine monitoring and targeted evaluation is designed to support the quality of services, systems, and governance. The M&E system prioritizes use of routinely collected national data supplemented by targeted additional data collection activities implemented as part of the PHIT Partnership on a set of key indicators that measure inputs, processes, outcomes, and selected impacts. These indicators include use of clinical areas at health facilities, facility readiness as documented by a rapid facility assessment, stockout of tracer drugs, and quality of care as observed by trained mentors.

The routine use of high quality data is vital for guiding decisions by facility managers and the intervention team. District hospital M&E staff are supported by a PHIT M&E intervention team during monthly health center visits for data quality assessments and interventions to address identified gaps. The system is designed to ensure that data are fed back to decision makers to facilitate evidence-based decisions. MOH staff are trained to use routinely collected data to determine health priorities, identify gaps for improvement, monitor health trends, evaluate programs, and identify areas for replication and dissemination.

### Component 3. Strengthened network of community health workers

A cornerstone of the Rwanda PHIT Partnership is the strengthening of the community health system based on a network of multidisciplinary CHWs. The emphasis on a strong network of CHWs reflects the Rwandan government’s recognition of the importance of decentralizing health care delivery to the community level as a means of encouraging communities to take responsibility for their own health, as well as promoting prevention activities, early detection of illness, and increased utilization of health services. The PHIT Partnership targets three major areas of the community health system: a strong supervision system to ensure CHW quality of care and reporting; implementation of a household register to improve quality through systematic documentation of household visit activities; and the expansion of home-based care provided for chronic disease patients, including those with HIV.

Under the national system, CHWs conduct monthly home visits to households in their village, providing health education, screening for specific diseases, encouraging health center visits, and monitoring child growth and development. During household visits and through routine community sensitization events, CHWs educate communities on key health messages, including hygiene and sanitation, accessing care at facilities, malaria prevention, and general health.

For logistical reasons, additional support to the community health worker network is being conducted in only one of the two intervention districts: southern Kayonza. CHWs in the intervention district receive ongoing training and supervision focused on primary health care, reproductive health, family planning, and malnutrition. During routine household visits, the CHWs receive regular supervision visits in which they are given feedback on quality of care delivered and on the quality of the data recorded during the visit. In each health center catchment area, CHWs belong to a cooperative that receives modest performance-based financial incentives.

### Project timeline

**Figure 4:**
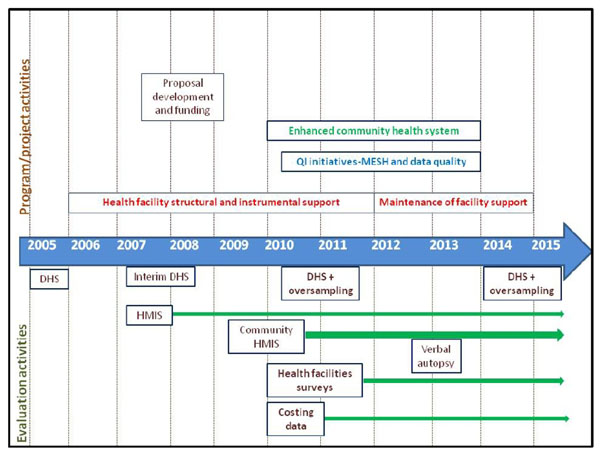
Timeline of implementation and evaluation activities.

The timeline of implementation and evaluation activities is presented in Figure 4.

### Implementation progress to date

By the end of the project’s second year (June 2011), all components of the PHIT intervention were successfully implemented. All 21 health centers and two district hospitals in the intervention area were adequately resourced, and the three core quality initiatives — the MESH program, joint M&E and data quality initiatives, and enhanced community health delivery — were operational. Based on early operational evidence of success, several intervention components are being evaluated by the Ministry of Health for potential integration into the national framework.

Key implementation challenges and subsequent adaptations are outlined in Table 3. While the overall implementation timeline has been followed, components of the intervention were refined over time to reflect changes in national policy, MOH priorities, district and community-based initiatives, as well as early operational research findings. Indeed, the rapidly evolving policy landscape — surely a factor in the national health sector’s success — has posed a challenge to implementing the intervention as designed.

**Table 3:** Rwanda PHIT Implementation progress: success, challenges, adaptations

*Successes*
• By the end of project Year Two (June 2011), all major components of the PHIT intervention were successfully implemented in the intervention area. • Based on encouraging early evidence, the Rwanda Ministry of Health is considering several components of the PHIT intervention for potential scale up. These include: o Mentorship and Enhanced Supervision (MESH) program to improve quality of care at health facilities o Community health household register to track activities and improve reporting o Electronic medical record for capturing patient visit data and generating alerts and reports to improve quality of care • Integrated operational research has been implemented within a number of PHIT intervention projects as part of the research agenda and a component of capacity-building activities initiated in partnership with the National University of Rwanda School of Public Health.

***Challenges & Adaptations***

• Although under-five mortality has decreased from 152 per 1,000 live births in 2005 to 76 in 2010, neonatal mortality has declined only marginally in the intervention area and countrywide.^1^ We are developing a care delivery value chain for neonatal health to identify gaps and respond accordingly. Incorporation of neonatal death reporting by community health workers and verbal autopsy will allow for real-time monitoring and rapid adaptation. • In 2012, the Ministry of Health implemented a policy to harmonize MOH staff salaries across the country. The loss of incentives has impacted staff retention in the intervention area. • Ensuring data quality and use of routinely collected data, while an anticipated challenge, has required more time and effort than originally planned.

^1^National Institute of Statistics of Rwanda (NISR) [Rwanda], Ministry of Health (MOH) [Rwanda], and ICF International.2011. **Rwanda Demographic and Health Survey 2010**. Calverton, Maryland, USA:NISR, MOH, and ICF International.

**Table 4:** Core components of the impact evaluation and operational research

	Focus	Data source	Analysis
Impact Evaluation	Impact and effective coverage	Oversampling DHS 2010 and 2015	Difference in differences in key population health indicators between 2010 and 2015, comparing the intervention area with rural comparison districts
	
	Service utilization and coverage, facility and community health worker levels	Routine HMIS from hospital, health centers and community health worker program	Change in service utilization and coverage in intervention areas in comparison to other rural districts
	
	Cause of death in children under five	Verbal autopsy through MOH program	Changes in the cause of death in children over the intervention period
	
	Contextual factors	DHS, National Resource Database, environmental records	Analysis of impact of contextual factors on district-level differences, including environmental factors, epidemics, humanitarian crises, sanitation, equity measures, women's education, HIV prevalence, Government of Rwanda records of partner contributions and engagement

Economic and Costing Analysis	Costing	Costing data collected by project	Economic and costing assessment of the health system in the intervention area including the costs per capita of PHIT intervention; total costs per capita of the health systems in the two intervention districts; and financial contributions made by government, partners and patients to the local health systems

Component Evaluation	Health facility support	Health facility survey, HMIS, MESH data	Impact of intervention on strengthening across WHO building blocks, explore relationship with quality and volume of care delivered
	
	MESH	External observation of care delivery, qualitative evaluation of district and health center staff	Effectiveness of MESH in improving quality of care focusing on children under-five, acceptability of and satisfaction with MESH
	
	Strengthened CHW systems	Community HMIS data, household register	Analysis of change in CHW-delivered services in intervention area and comparison areas
	
	Operational research	Project specific	Includes assessment of PHIT intervention on family-planning uptake, human resource retention, nutritional status in children

## Evaluation design

The PHIT implementation program is complemented by a systematic evaluation designed to 1) measure the population health impact of the district-based health systems strengthening intervention; 2) assess the total cost per capita of the health system in the intervention area; 3) study the efficacy of component interventions, including targeted instrumental support to health facilities, the MESH quality improvement program, strengthening of M&E and data quality systems, and the enhanced community health program; and 4) conduct operational research on the implementation of the program (Table 4).

### Overall impact evaluation

The impact evaluation includes four major components: an analysis of the difference in differences in key population health indicators between 2010 and 2015; assessment of trends in routinely collected indicators from the national HMIS; assessment of changes in the causes of death in children measured through verbal autopsies; and analysis of contextual factors that may impact district-level differences.

#### Analysis of DHS data

We are measuring temporal changes in coverage, health indicators, and contextual factors through the routine DHS conducted in 2010 and 2015, and comparing these differences in the intervention area to those in other rural areas in Rwanda. The DHS are standardized household surveys that use a cluster sampling strategy to generate representative population, health, and nutrition indicators at the national and sub-national (DHS Region) level for monitoring and evaluation activities [18].

In both the baseline and final survey, we oversample our intervention area using national DHS methodology [19] to collect an adequately powered sample to estimate difference-in-differences in under-5 mortality and other PHIT collaborative core indicators between the intervention and rural control areas from 2010 to 2015. We will conduct a hierarchical regression analysis that includes the PHIT intervention as one of multiple province- and district-level variables expected to impact under-5 mortality.

The DHS data will also provide information on changes within the districts on other key indicators of population health, including stunting, wasting, and total fertility rate. The data are also the source for information on health access indicators: contraceptive prevalence, unmet need for family planning, antenatal care utilization, treatment of childhood illness, and immunization coverage.

#### Trends in facility and community health HMIS data

Analysis of routinely collected HMIS data will complement the DHS analysis in two ways. First, comparison of key indicators assessed through DHS and HMIS may mitigate the potential heterogeneity of DHS data when used for sub-national analysis [20]. Second, HMIS data will be used to assess PHIT collaborative indicators not collected by the DHS.

Data from the community health center and district hospital levels are aggregated and submitted monthly by districts to a national HMIS database. We will analyze trends in service delivery and coverage at facility and community levels, comparing key HMIS-derived indicators before, during, and after the start of the intervention. HMIS data will also be used to measure trends in service volume and selected process indicators not captured in DHS, comparing intervention area facilities with those in the rural control area.

#### Verbal autopsy

The MOH has initiated a national program of routine verbal autopsy (VA) for all maternal and child deaths and is currently implementing this approach nationally, including in the intervention area. The PHIT intervention supports this effort by developing and implementing trainings in VA, and by introducing innovative methods for analyses of VA data. VA will allow for more precise analysis of trends in under-5 deaths and gain a better understanding of under-5 mortality data, which may have important programming implications.

#### Exogenous and contextual factors

In order to control for potential external confounders, we are assessing exogenous factors expected to have an impact on population health and selected service delivery indicators. We are working with the relevant ministries to develop a database to track such catastrophic events as epidemics and humanitarian crises. Other relevant contextual factors will include sanitation, equity, women’s education, and HIV prevalence, all of which are available through the DHS. These exogenous factors will be included as potential confounders in our regression modeling of district level health outcomes.

### Economic and costing analysis

The main purpose of the economic analysis is to understand how the PHIT program contributes to local health system financing. The evaluation measures 1) the costs per capita of PHIT investment in each of the six WHO health system building blocks; 2) total cost per capita of the health system in the two intervention districts; 3) health facility spending by WHO building block; and 4) financial contributions to the local health system made by government, the PHIT Partnership, other partners, and patients. We have designed survey instruments for each facility type to measure funding sources, facility expenditure, and existing capital. Cost data have been collected for the baseline year (2010) and the surveys will be repeated annually through 2014.

We will conduct trend analyses in key variables over time, including the level and trends of total health system and PHIT spending per capita in the two districts; percentage of total health expenditures contributed by PHIT, government investment, and other sources; and the level and trends of expenditure on each of the six WHO building blocks by funding source. We will use regression analysis to evaluate the impact of PHIT spending on health care provision using the provision of child health services as the outcome variable.

### Component evaluations of the PHIT intervention

Operational evaluations will focus on key components of the PHIT intervention — targeted instrumental support to health facility building blocks, quality improvement through the MESH program and strengthened M&E systems, and enhancements to the community health system. These evaluations will serve two purposes: use of the data for modification and improvement of the interventions throughout the implementation period and exploration of the relative contributions of each component intervention on health outcomes to inform replication and scale-up of the Rwanda PHIT intervention.

#### Health facility instrumental support

As described above, a standardized health facility survey has been developed and administered before the initiation of the health center instrumental support, with plans to re-administer quarterly. Changes in domain scores and overall facility scores will be tracked and compared over time. Differences in achieving fully capacitated and high-quality systems across the sites will be explored to identify factors associated with more robust or rapid success and to identify areas where the strategy has been less effective. Multivariate models will adjust for potential confounders including site size, duration of PIH support, and remoteness of health facilities.

#### Mentorship and enhanced supervision (MESH) program evaluation

The evaluation of the MESH program will measure the change in quality of care delivered at the health center level. The main evaluation focuses on quality of child health care delivered by MESH-supported nurses and the primary outcome is the integrated management of childhood illness (IMCI) integrated assessment index, derived from a checklist of protocol-defined assessment tasks that should be performed for all children [21]. Secondary outcomes include individual assessment components, classification, treatment, and IMCI coverage indicators.

#### Assessment of data quality and utilization

Because the validity of analyses using routinely reported data depends on the quality of the data collected, we are studying the reliability of CHW data using Lot Quality Assurance Sampling (LQAS). LQAS allows for rapid classification of CHW reports as having high or low quality, defined as concordance between reports aggregated at the health center level and the original registries. This approach will provide a rapid and inexpensive methodology to target data quality improvement efforts and measure their impact [22].

We will also measure changes in data quality of standard health facility reports. Work is ongoing to further develop mixed method evaluation of change in capacity for data interpretation, utilization of resources to address identified gaps, self-reported knowledge in data management, and interpretation.

#### Assessment of the strengthened network of community health workers

Using routinely collected data from monthly community HMIS reports, we will assess health indicators from intervention area sectors compared to other rural sectors. In a second approach, we will leverage an ongoing World Bank-sponsored study that randomized 200 community health cooperatives (all CHWs in a health center catchment from one cooperative) to one of four different incentive models by applying its results to our interventions. We will, thus, pool data from the intervention area cooperatives and compare them to the non-PHIT cooperatives adjusting for the intervention arm assigned.

### Research capacity building through operational research

In order to address a critical skills gap in Rwanda, an operational research capacity building effort has been embedded within the PHIT evaluation plan. Led by National University of Rwanda School of Public Health and Harvard Medical School, we launched an MPhil and PhD research training program, known as Rwanda PHIT Scholars, for 10 Rwandan health professionals involved in the PHIT Partnership. Candidates underwent a competitive selection process that involved the development of operational research proposals to complement the PHIT impact evaluation and intervention component evaluations. Each student has been assigned an advisory team, including a NUR-SPH advisor, an HMS faculty advisor, and a PHIT Rwanda site supervisor.

Among the student-led operational projects are assessments of family planning uptake, performance of the electronic medical record (EMR) currently under development by the PHIT Partnership, the impact of Rwanda’s e-health architecture, the impact of HIV care delivery on maternal and child health services, the impact of the PHIT intervention on childhood nutritional status and health facility staff retention, and ethnographic assessment of family planning utilization.

## Discussion

In the context of global efforts to achieve the health-related Millennium Development Goals, there is a strong and growing consensus that evidence-based interventions are needed to create “robust, responsive, and efficient health systems” [23]. We believe that adhering to the five principles of the Rwanda PHIT Partnership while focusing HSS on all the levels of the district health system are essential to reaching this goal.

Health systems strengthening interventions should be *comprehensive*. Much as vertical, or disease-specific, interventions may paradoxically weaken primary health systems [24], narrowly focused HSS interventions may limit value by neglecting other gaps in the health system. For example, a robust initiative to recruit and train health workers is unlikely to succeed if those health workers are asked to perform in a setting of decrepit infrastructure, inadequate equipment, drug stock outs, and absent information systems. For this reason, our intervention deliberately emphasizes capacitation across all six WHO building blocks at the district, facility, and community levels of the health system.

Second, HSS interventions should be *integrated*, adopting a “systems thinking” approach [13]. Such an approach takes into consideration that changes in one building block of the health system are likely to affect other building blocks. Thus, our three main intervention components have been designed to work across multiple health system building blocks and at all levels of the district health systems.

Third, HSS interventions should be *locally responsive*; that is, guided by both national priorities and the needs of an engaged community. The WHO Health Systems Framework acknowledges that health systems are highly context-specific and that systems strengthening interventions need to be developed accordingly [25]. Emphasizing local participation, we developed a two-step approach that employed multidimensional health systems gap analyses to inform evidence-based and participatory resource allocation, thus ensuring that health systems remain responsive to local needs. Community health advisory boards ensure that implementation is continually responsive to the local context.

Fourth, in order to create sustained value, HSS interventions must *strengthen institutions*. Dr. Julio Frenk, Dean of the Harvard Medical School, highlights the importance of cultivating strong ministries of health, which can only be accomplished through long-term investment in the public sector [26]. Our intervention emphasizes joint planning and work to support and strengthen local governance and leadership to reinforce district and facility-level public institutions.

Fifth, HSS interventions should address *access*, *quality*, *and cost* issues. Universal access to health services is both an equity issue and a sound public health principle. In Rwanda, efforts to provide universal community-based health insurance — an initiative supported by the PHIT Partnership —are already bearing fruit [7]. This final principle is also core to the three main components which all focus on ensuring accessible quality care.

While basic health infrastructure and systems inputs may be a prerequisite for quality improvement efforts, there is growing recognition that QI is a key to improved efficiency and health outcomes in global health systems [27,28]. The PHIT intervention extends QI efforts beyond clinical service delivery in health facilities, targeting integrated health information systems, supply chain, and governance at multiple levels of the health system.

Given the financial constraints experienced by both developing country governments and external funders in a global economic downturn, the importance of smart resource allocation and value creation — defined as health outcomes per dollar spent — is difficult to overstate [29]. The Rwanda PHIT evaluation emphasizes this principle by strengthening evidence-based resource allocation, and integrated routine monitoring and evaluation to identify areas where additional effort is needed. The exhaustive economic and costing analysis will map inputs across building blocks and health system levels, and facilitate comparative value analysis with other health system interventions. We expect these findings to contribute to future decision making about scale-up of various components of the PHIT intervention.

There is a critical need to promote health systems impact assessments to generate a stronger evidence base on what works in resource-constrained health systems [30]. Only an impact evaluation designed to measure the full spectrum of interventions, as well as the cost and external factors, will be able to provide the critical information needed by implementers, governments, and donors to make effective choices in efforts to improve population health through HSS. By combining decades of implementation experience with continuous experimentation and a robust impact evaluation, we believe that the Rwanda PHIT Partnership is poised to generate replicable lessons that can improve health and health equity in Rwanda and throughout the developing world.

## List of abbreviations used

BWH: Brigham and Women’s Hospital; DHS: Demographic health survey; EMR: Electronic medical record; HMIS: Health management information system; HSS: Health systems strengthening; HSSP-II: Health Sector Strategic Plan; IMAI: Integrated management of adult illness; IMCI: Integrated management of childhood illness; LQAS: Lot quality assurance sampling; M&E: Monitoring and evaluation; MESH: Mentorship and Enhanced Supervision of Health Centers; MOH: Ministry of Health; NISR: National Institute of Statistics of Rwanda; NUR-SPH: National University of Rwanda-School of Public Health; PHIT: Population Health Implementation and Training; PIH: Partners In Health; QI: Quality improvement; VA: Verbal autopsy; WHO: World Health Organization.

## Competing interests

The authors declare that they have no competing interests.

## Authors’ contributions

All authors have made substantial contributions to conception and design, have been involved in drafting the manuscript or revising it critically for important intellectual content, and have given final approval of the version to be published.

## Declarations

This article has been published as part of *BMC Health Services Research* Volume 13 Supplement 2, 2013: Improving primary health care to achieve population impact: the African Health Initiative. The full contents of the supplement are available online at http://www.biomedcentral.com/bmchealthservres/supplements/13/S2. Publication of this supplement was supported by the African Health Initiative of the Doris Duke Charitable Foundation.
